# COSMOS—improving the quality of life in nursing home patients: protocol for an effectiveness-implementation cluster randomized clinical hybrid trial

**DOI:** 10.1186/s13012-015-0310-5

**Published:** 2015-09-15

**Authors:** Bettina S. Husebo, Elisabeth Flo, Dag Aarsland, Geir Selbaek, Ingelin Testad, Christine Gulla, Irene Aasmul, Clive Ballard

**Affiliations:** Department of Global Public Health and Primary Care, Centre for Elderly – and Nursing Home Medicine, University of Bergen, Kalfarveien 31, N-5020 Bergen, Norway; Centre for Elderly and Nursing Home Medicine, Stavanger University Hospital, Stavanger, Norway; Karolinska Institutet (KI), Department of Neurobiology, Care Sciences and Society, KI-Alzheimer Disease Research Center, Stockholm, Sweden; Norwegian National Advisory Unit of Ageing and Health, Vestfold Hospital Trust, Tønsberg, Norway; Centre for Old Age Psychiatry Research, Innlandet Hospital Trust, Ottestad, Norway; Institute of Health and Society, Faculty of Medicine, University of Oslo, Oslo, Norway; The Wolfson Wing & Hodgkin Building Guys Campus, Kings College, London, SE1 1UL UK

## Abstract

**Background:**

Nursing home patients have complex mental and physical health problems, disabilities and social needs, combined with widespread prescription of psychotropic drugs. Preservation of their quality of life is an important goal. This can only be achieved within nursing homes that offer competent clinical conditions of treatment and care. *CO*mmunication, *S*ystematic assessment and treatment of pain, *M*edication review, *O*ccupational therapy, *S*afety (COSMOS) is an effectiveness-implementation hybrid trial that combines and implements organization of activities evidence-based interventions to improve staff competence and thereby the patients’ quality of life, mental health and safety. The aim of this paper is to describe the development, content and implementation process of the COSMOS trial.

**Methods/Design:**

COSMOS includes a 2-month pilot study with 128 participants distributed among nine Norwegian nursing homes, and a 4-month multicenter, cluster randomized effectiveness-implementation clinical hybrid trial with follow-up at month 9, including 571 patients from 67 nursing home units (one unit defined as one cluster). Clusters are randomized to COSMOS intervention or current best practice (control group). The intervention group will receive a 2-day education program including written guidelines, repeated theoretical and practical training (credited education of caregivers, physicians and nursing home managers), case discussions and role play. The 1-day midway evaluation, information and interviews of nursing staff and a telephone hotline all support the implementation process. Outcome measures include quality of life in late-stage dementia, neuropsychiatric symptoms, activities of daily living, pain, depression, sleep, medication, cost-utility analysis, hospital admission and mortality.

**Discussion:**

Despite complex medical and psychosocial challenges, nursing home patients are often treated by staff possessing low level skills, lacking education and in facilities with a high staff turnover. Implementation of a research-based multicomponent intervention may improve staff’s knowledge and competence and consequently the quality of life of nursing home patients in general and people with dementia in particular.

**Trial registration:**

ClinicalTrials.gov NCT02238652

## Background

The rapidly growing population of elderly persons in Europe is subject to frequent and numerous comorbidities, impaired organ function and problems linked to access to care and skilled treatment [1]. Dementia is increasingly common in the ageing population, with approximately 35 million affected people worldwide and 10 million in Europe [2, 3]. This number is expected to double within the next three decades, thus posing a considerable challenge for the healthcare system and society. Whereas one in four Americans die in a nursing home (NH) every year [4], almost half of the Norwegian citizens die in a NH [5]. In Norway, it has been estimated that 70,000 people have dementia, 34,000 of whom live in a NH [6] and have a stay of about 24 months mean length before death. More than 80 % of those living in a NH have dementia [7], often combined with stroke, heart failure or cancer. They have distressing mental health problems, such as agitation and depression [8], physical disabilities and unmet social requirements, and they are often in pain [9, 10]. The prescription of medication is high, including potentially harmful psychotropic drugs [11]. In addition, these people are in significant need of advance care planning [12] and meaningful activities [13].

In order to meet these challenges, the Norwegian Government encourages the municipalities to develop services and staff competence to improve mental health and quality of life (QoL) in NH patients and people with dementia as set out in the Coordination Reform (Norwegian Government report: 47 2008-2009). Objectives are in line with the National Research Program on Health, Care and Welfare Services 2015–2024 supporting research to develop and evaluate effective and complex interventions in large-scale research projects with a multidisciplinary approach, and including elderly NH patients with chronic diseases.

Responsibility for care and treatment of older people depends on the commitment and capability of the primary healthcare system. However, despite these complex tasks, NH patients are often treated by unqualified staff who lack education, knowledge and basic skills in terms of understanding patient behaviour, and who have insufficient expertise in how to treat and give proper care to persons with dementia [6].

### Rationale for the present trial

The number of cluster randomized clinical trials (RCT) including NH patients with and without dementia that are designed to investigate the efficacy of competence improvement programs combined with clinical treatment methods has increased in the last decade. For instance, implementation of introductory communication in the form of advance care planning (ACP) in NHs resulted in fewer deaths in hospitals and reduced resource use [12], better end-of-life care and pertinent ethical discussions, and satisfied relatives and staff [14]. Another example is the stepwise protocol of treating pain (SPTP) in people with dementia which succeeded in the reduction of agitation [15], mood syndrome [16] and pain [10]. Previously, systematic medication reviews, including staff education, workshops and face-to-face interaction between the prescribing physician and an expert-group, have been found to reduce unnecessary and harmful drug prescription [17]. Finally, a current systematic review by Testad et al. (2014) highlighted the benefit of systematic organization of activities [13] and described improvements in neuropsychiatric symptoms for reminiscence therapy [18–20], personalized pleasant activities [21–23] and person-centered care [24–26].

It is of key importance that these single interventions improved either behaviour such as agitation and aggression, reduced use of the total medication and antipsychotics, or hospital admissions. However, none of the single interventions resulted in an improvement of the QoL in NH patients. In addition, the evaluation of the actual implementation process of the interventions has largely been neglected.

### The COSMOS intervention

**Fig. 1:**
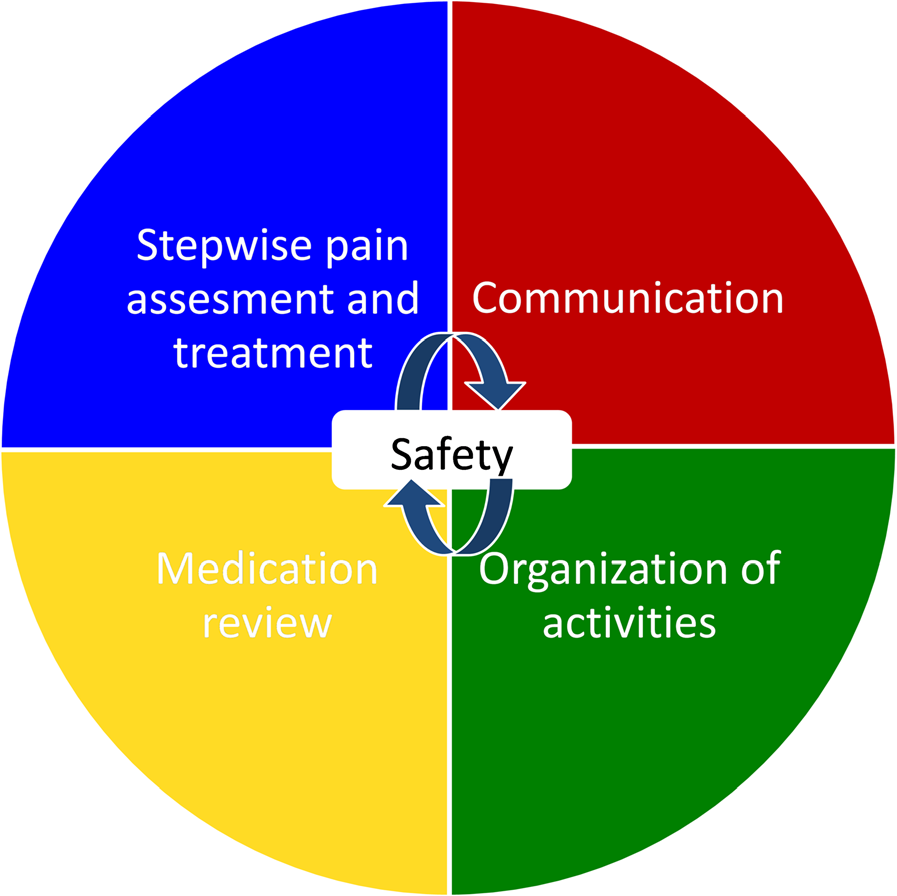
COSMOS components

It is a basic requirement of human rights that a person is informed about her or his disease and be enabled to consider future plans and decisions [27–29], be out of pain [15, 30, 31], receive proper medical treatment [32, 33] and be involved in meaningful activities [13, 21, 34]. The COSMOS intervention is based upon results of earlier RCTs and will combine, implement and test the most effective components for developing an optimal multicomponent and systematic intervention by (Fig. 1):

- *CO*mmunication
- *S*ystematic assessment and treatment of pain
- *M*edication review
- Organization of activities and
- *S*afety

Changing clinical practice requires attention to multiple factors that influence individuals’ or groups’ willingness and ability to incorporate new knowledge of care [35]. Whereas education of clinicians alone is reported to be ineffective in changing care practices, complex multicomponent interventions that incorporate clinicians’ education have been reported to be successful [36]. The COSMOS trial is an evidence-based effectiveness-implementation hybrid trial funded by the Norwegian Research Council. We intend to use mixed qualitative and quantitative methods to test the effectiveness of the multifaceted intervention and to develop and test the implementation strategy for NH staff. In this paper, we describe the development of the COSMOS intervention; development of the education program; education of the COSMOS implementers (COSMOS ambassadors); implementation process in intervention NHs; support procedures, and statistical analyses.

The COSMOS program is based on evidence from the literature, and scientific and clinical experiences of the research group including research projects with cross-sectional, trajectory and RCT design, and a review of the literature. The COSMOS researchers are involved in education programs and teaching activities for healthcare professionals, people with dementia and relatives both at a national and international level. The combination of evidence for best practice and expertise related to education and training has been used to build up a systematic intervention which transfers evidence-based knowledge into an understandable everyday quality improvement intervention.

### Implementation strategies

One of the greatest challenges facing the global health community is to take proven interventions and implement them into the real world. The term “to implement” means “to carry out into effect”. Implementation research is defined as the related scientific investigation concerning the implementation process and the act of carrying an intention into effect in a real-world scenario [37, 38]. For the COSMOS trial, this means that research-based knowledge is to be transferred into practice with the selection of NH patients who mirror a broad variety of current quality in care and treatment offered in NHs.

A crucial aspect when assessing the effect of a complex intervention study is whether or not the intervention was implemented at all. Even when an intervention is superbly designed, real-world contextual factors may prevent the intervention from being realized as intended in a complex adaptive system [39, 40]. The intervention may not be completed, or it may be completed differently than originally intended, not systematic or plainly wrong. In other words, it is necessary not only to evaluate the intervention effect but also to evaluate implementation fidelity and sustainability [40].

### Aims of the COSMOS trial

The primary objective with the COSMOS trial is to improve the QoL in NH patients by enhanced communication in form of ACP, proactive assessment and treatment of pain, discontinuation of unnecessary medication and organization of activities. The secondary objective is to determine the effectiveness by core outcome measurements of mental and physical health, pain, sleep, safety, total drug use, hospital admission and cost-effectiveness. We also investigate how successful the implementation process was and staff satisfaction.

## Methods and design

**Fig. 2:**
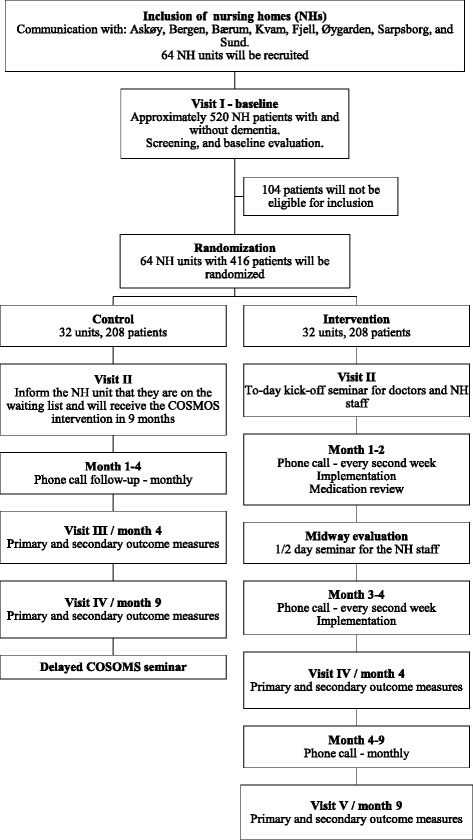
Flow chart of the COSMOS trial

This is a 4-month multicenter, cluster randomized and controlled effectiveness-implementation hybrid trial with follow-up at month 9, involving 571 participants from 67 NH units in Norway (Flow chart in Fig. 2). The mixed method design comprises the quantitative assessment with validated outcome measures and qualitative research of the intervention strategy by implementation indicators [40]. Very few studies have focused on these critical issues, hence the key attention of this project is to explore how the combination of an educational program for carers and research-based practice and competence may improve the QoL for NH patients. Until now, the efforts to improve standard of care in NHs have resulted in many “stints”; that is, most NHs may have had a campaign focusing on one of the single interventions. However, if a NH, for instance, offers optimal assessment and treatment of pain, this does not automatically mean that patients are also provided with meaningful activities.

### Settings and target population

Urban and rural NHs in Southern Norway will be included. The mix between urban/rural, big/small municipalities from different areas of Norway, extending over 700 kilometers apart, ensures a representative NH population. Systematic selection of the clusters will be achieved through established networks and information to related municipalities and NH managers. We strived to ensure collaboration from the top healthcare leaders of each municipality.

Inclusion criteria: NH patients with and without dementia, ≥65 years old, from 67 clusters, will be recruited. Diagnoses are based on patient’s medical records, medical examination and routine laboratory tests in the NH. The patients’ cognitive function is assessed by Mini-Mental State Examination (MMSE) [41] and Functional Assessment Staging (FAST) [42].

Exclusion criteria: Dying patients (life expectancy ≤6 months) or patients with diagnoses of schizophrenia will not be included.

### Research questions and study hypotheses in order to meet the aims of the study

1. To what extent will the implementation of ACP improve the decision processes and interactions between patients, staff and family and reduce hospital admissions and costs? We hypothesize that a systematic communication approach will empower patients and families to make preferred choices, be more independent and become better able to understand the complexity of current diagnoses, care and treatment.
2. To what extent will previously unidentified pain be uncovered when the use of MOBID-2 is implemented? In patients with untreated pain, to what extent will a stepwise protocol of treating pain, show benefit on self-reported or proxy-rater assessed pain by MOBID-2 Pain Scale? We hypothesize that education and written material will result in improvements in pain assessment which will in turn result in excellent pain treatment and improved QoL.
3. In patients with polypharmacy (≥4 drugs), to what extent will the systematic protocol of medication review based on face-to-face discussion between the responsible physician, NH staff and research team, following START and STOPP criteria [43] show benefits in terms of total medication use, use of psychotropic drugs and costs [44]. We hypothesize that a systematic medication review will reduce unnecessary medication and related costs, thereby improving the resources available.
4. To what extent will a standardized and individual plan for activities increase activity time? We hypothesize that an individual plan for activities will improve the daytime activity provision to patients through regular follow-up and inclusion of relatives and volunteers.

### Cluster randomized effectiveness-implementation hybrid trial

Whereas pragmatic trials conduct a fixed intervention and do not try to control or ensure the delivery of services to meet realistic standards in normal practice settings, effectiveness-implementation hybrid trials also intervene and/or observe the implementation process as it actually occurs [37, 45]. Thereby, effectiveness-implementation hybrid designs are intended to assess the effectiveness of both an intervention and an implementation strategy. In this context, the expression “hybrid” signalizes a mixed method study design to cover the whole process of implementation and assessment of the intervention. Studies include elements of an effectiveness design (e.g. randomization to intervention and control group) and investigate, additionally, the implementation strategy by implementation outcome variables [45].

#### Cluster design and blinding

According to the research design, patients, units or even NHs are cluster randomized to care as usual or treatment. In a hybrid trial design, the implementation strategies, education and follow-up may be optimized during the process, for the purpose of gaining new understanding and insight. Because of this, the participants, patients and/or staff related to the clusters cannot be completely blinded regarding the group allocation. Meanwhile, the cluster randomized design is the most suitable design for implementation research, as it reduces the contamination between the intervention and control groups [46]. Furthermore, the cluster design takes into account the fact that the participants live together in the NH clusters.

#### Randomization

Using SPSS, each single NH unit is randomized to intervention or control condition per participating municipality and matched by urban and rural, prosperous and less well-to-do status and organizational conditions.

### Control condition

The control group will receive care as usual, during the trial and follow-up period. NHs in the control group have to show a satisfactory standard of care. This standard will be verified by the COSMOS team during the data collection. In addition, the control group will be monitored by monthly telephone contact. The control group may also derive a considerable learning effect. Before randomization, representatives from each NH receive information about the aim and content of COSMOS, because they have to decide whether they are interested in participating or not. When NHs are then randomized for control, responsible staff members receive information on dementia diagnoses, neuropsychiatric symptoms and pain assessment in people with dementia as part of the data collection, and they will be trained in the use of primary/secondary outcome measures. To motivate control NHs to continue participating, they will receive the COSMOS intervention after the last data collection at month 9 by a waiting-list-strategy [47] supported by a “supervisor” recruited from a NH who has already received the intervention.

### Development of the COSMOS education program

**Fig. 3:**
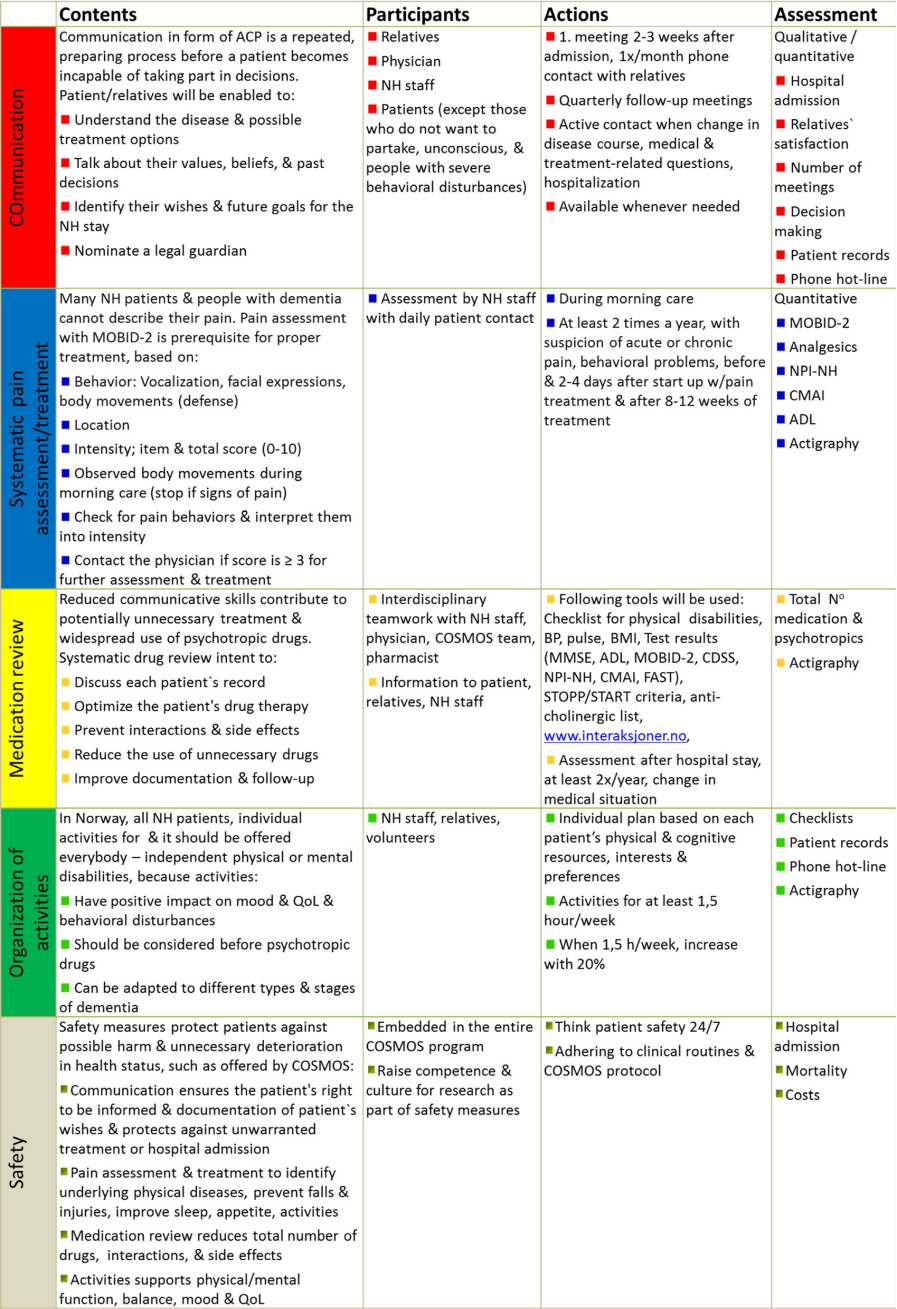
Detailed overview of the multicomponent COSMOS intervention, education program and outcome measures

**Fig. 4:**
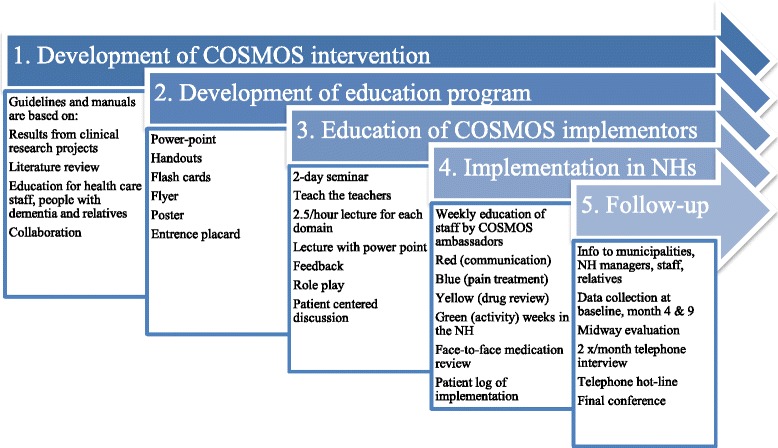
COSMOS protocol development and implementation strategy

The study intervention will be delivered by the COSMOS education program and guidelines describing the COSMOS components: *CO*mmunication, *S*ystematic assessment and treatment of pain, *M*edication review, Organization of activities, and *S*afety (Figs. 3, 4 and Appendix). Standardized written material (guidelines, patient logs, power point, handouts, flash cards, flyer, poster and entrance placard) that describe the evidence base background and content of COSMOS is prerequisite for training. The material has been adapted to language and staff competence and reviewed taking into account mental health and care needs of the patients. After the pilot study, adaptions are made for the power point presentation, time use and enhanced feedback system, whereas the content of the intervention is changed only marginally. For further follow-up, the standardized and pilot-tested 2-day teaching program (Appendix) will transfer COSMOS components (Fig. 3) to the implementers (COSMOS ambassadors), by a senior researcher (BSH) and postdoctoral research fellow (EF). Selected NH staff, NH physicians and managers are invited; at least two colleagues from included units must participate.

### Education of the COSMOS ambassadors

The intervention will be delivered by implementers called “COSMOS ambassadors”. The COSMOS ambassadors are staff from the intervention NHs. We invite all physicians, nurses and licensed practical nurses to the education, but require a minimum of two nurses from each NH unit in the intervention group to participate. The COSMOS ambassadors usually registered and licensed practical nurses have hands-on experience with NH patients in their daily work. Ambassadors participate in the 2-day education (about 2.5 h per COSMOS component, role play, patient-centered discussion and feedback) following the COSMOS program (Fig. 3 and Appendix) and receive supervision from the research team using the COSMOS patient logs and written COSMOS guidelines. NH managers provide a written agreement for participation and confirm that staff will not switch between care units. Given the complexity and multi-faced nature of the intervention, as well as the heterogeneous “real-world” population, some variability in the implementation of the interventions is to be expected.

### Implementation in NHs

After finalizing the COSMOS education program, the intervention (Figs. 3 and 4) will be delivered by the ambassadors at each NH unit with a weekly focus; “red week” for communication, “blue” for systematic assessment and treatment of pain, “yellow” for medication review and “green” for organization of activities. Education will be offered during lunch and/or report for about 20 min, if possible, several times a week to enable all staff members to participate. The ambassadors will use written material and power point presentation to inform and educate their colleagues. By this, each COSMOS component will ideally be repeated every month between baseline and month 4 data collections. To ensure medication review, two COSMOS researchers (BSH and CG) sought out the NH physicians and responsible nurses to perform a collegial face-to-face systematic medication review. The support by regular telephone contact every second week, a telephone/email hotline (Monday to Friday 08:00–16:00) gives NH staff assistance when they have concrete questions related to data collection or internal education. A half day, midway evaluation after 2 months, and personal visits if requested by staff members, is offered to further facilitate implementation. The COSMOS ambassador at each intervention NH will supervise the overall delivery of the interventions, supported by four full-time COSMOS researchers (IA, CG, TH and TE).

### Effectiveness measures by core outcome measurements

Data collection for outcome measures will be completed at baseline, months 4 and 9, conducted by the patient’s primary caregiver who knows the patient, together with a research assistant. Demographics will be collected from the patients’ record. Selection of outcome measures is consistent with recommendations from the Initiative on Methods, Measurement and Pain Assessment in Clinical Trials (IMMPACT) [48].

### Primary and secondary outcome measures

The primary outcome in the COSMOS trial is QoL as measured by quality of life in late-stage dementia (QUALID) [49] and Quality of life in Dementia (QUALIDEM) [50] (Table 1). We also use the European Quality of Life-5 Dimensions (EQ-5D) [51] in connection with Resource Utilization in Dementia-Formal Care (RUD-FOCA) [44] as one of the secondary outcomes. Other secondary outcomes are Neuropsychiatric Inventory-Nursing Home version (NPI-NH) [52], Cohen-Mansfield Agitation Inventory (CMAI) [53], Cornell Scale for Depression in Dementia (CSDD) [54], Mobilization-Observation-Behaviour-Intensity-Dementia 2 (MOBID-2) Pain Scale [55, 56], Personal Activities of Daily Living (P-ADL) [57] and Clinical Global Impression of change (CGIC) [58, 59]. We will use Actigraphy (Philips Actiwatch Spectrum) to objectively assess sleep patterns and circadian rhythm and light exposure [60, 61]. Total medication and use of psychotropic drugs in number and dose will be assessed with respect to drug-related problems and drug–drug interactions using STOPP and START criteria [43] and anticholinergic list [43]. A full description of the primary and secondary outcomes and screening instruments MMSE [41] and FAST [42] are provided in Table 1.

**Table 1:** Instruments used as primary and secondary outcome measures

	What does the tool measure	Tool characteristics & psychometric properties
QUALID*† [49]	QoL by cognitive function, health, function, social and psychological well-being.	11 behaviours rated on a 5-point Likert scale (range 11–55). Excellent reliability, internal consistency and validity are reported. Lower score indicates higher QoL
QUALIDEM*† [50]	QoL by self-image, affect, restlessness, care and social relation, feeling at home & active.	40 items scored 0–3 in 10 subscales yielding a sum score for each subscale; care relationship (0–21), positive affect (0–18), negative affect (0–9), restless tense behaviour (0–9), positive self-image (0–9), social relations (0–18), feeling at home (0–12), having something to do (0–6), undefined items (0–9). Sufficient reliability and validity are reported
EQ-5D*† [51, 69]	QoL by mobility, self-care, activities, pain/discomfort and anxiety/depression, and impression of health	Patient or care-giver indicates patient`s state in f the 5 dimensions, according to 3 levels: no, some or extreme problems, and total impression of health (0–100). Scarce evidence for use in NH setting & with/in people with dementia
NPI-NH*† [52, 70]	Neuropsychiatric symptoms in dementia, caregiver distress.	Total and subscale scores are provided based on frequency & severity of symptoms (range 0–144). Good validity and reliability of the Norwegian version of the NPI-NH. Including The neuropsychiatric inventory caregiver distress scale
CMAI*† [53, X7]	Agitation & behavioural disturbances	29 items (range 29–203). Good validity & reliability
CSDD*† [54]	Depression in people with dementia	19 items rated from 0=no symptom to 2=severe. ≥8 = depression, >12=moderate-severe depression. Satisfactory inter-rater reliability and validity
MOBID-2 Pain Scale*† [55]	Two-part pain location and intensity in people with advanced dementia.	Pain intensity inferred by the patient’s pain behaviours during standardized, guided movements (Part 1), and pain behaviours related to internal organs, head and skin (Part 2). Excellent reliability, validity and good responsiveness
MMSE † [71]	Differentiation of severity of cognitive impairment	30-point scale where 0 to 11=severe impairment, 12 to 17=moderate, 18 to 23=mild, 24 to 30=no impairment
FAST*† [42, X14]	Severity of dementia	Stages dementia in 7 stages, 1 normal, 2 normal ageing, 3 possible dementia, 4 mild, 5 moderate, 6 and 7 severe dementia. Good reliability and validity
ADL*† [57]	Physical function by rating activities; feeding, moving, toilet and dressing.	The scale includes 6 items (range 0–30) Lower values indicates better functioning and independence
CGIC* [72]	Perceived improvement and efficacy	7-point rating ranging from very much worse (0) to very much improved (6). Not intended as a sensitive measure of small changes, but for changes considered clinically significant.
RUD-FOCA* [44]	Cost-analysis of time use during 24 hours	Total time per 24 hours is summed and mean time is calculated by records of required care. Validated for use in NHs, acceptable test-retest reliability and construct validity

* Proxy rated instrument, † Validated for use in people with dementia, *ADL* Physical Self-Maintenance Scale, *FAST* Functional Assessment Staging, *CGIC* Clinical Global Impression of Change, *CMAI* Cohen-Mansfield Agitation Inventory, *CSDD* Cornell Scale for Depression in Dementia, *EQ-5D* European Quality of Life-5 Dimensions, *MMSE* Mini Mental State Examination, *MOBID 2* Mobilization-Observation-Behaviour-Intensity-Dementia 2 Pain Scale, *NPI-NH* Neuropsychiatric Inventory- NH version, *QoL* Quality of life, *QUALID* quality of life in late-stage dementia, *QUALIDEM* Quality of life in Dementia, *RUD-FOCA* Resource Utilization in Dementia – Formal Care

### Indicators of effective implementation strategies (qualitative)

The interventions will be observed and evaluated according to i) perception among end users and stakeholders that the intervention is “agreeable” (Acceptability); ii) staff intentions and actions to employ the intervention (Adoption); iii) perceived relevance of the intervention in NH settings (Appropriateness); iv) degree to which the intervention can be carried out in NHs (Feasibility); v) integrity to and quality of intended program delivery (Fidelity); vi) extent of institutionalization of the interventions, reach or spread (Penetration); vii) maintenance and continuation of the interventions; durability; integration; incorporation (Sustainability) [62, 63]. The evaluations include analyses of medical records and interviews of staff. Assessment of the implementation of the COSMOS interventions will be completed with monthly visits and phone calls to the contact persons on each NH. The patient logs will help structure the phone conversations according to individual intervention and patient. These semi-structured interviews will be coded in accordance with the patient log. This entails registering whether or not the planned interventions have been carried out (yes/no/not applicable), and collecting short statements regarding barriers and other relevant comments.

### Data management and analyses

A data manager will be responsible for punching, validating and merging trial data. Data will be stored on approved servers at the University of Bergen (UoB). Demographic and clinical characteristics between intervention and control at baseline will be compared using Pearson *χ*^2^ test statistics for categorical variables and independent samples *t* test for normal variables (age, diagnoses and pain diagnoses). Analysis of covariance (ANCOVA) estimates the mean effect in each trial arm, weighted across clusters (1 cluster = 1 NH unit) according to number of patients within each cluster, and from this, the mean treatment effect is estimated at each time point [47]. The intraclass correlation coefficient (ICC) expresses the proportion of the total variance in data between included clusters. Primary efficacy population includes all patients with at least one post-baseline assessment (month 4 measure), and we will use a linear intercept mixed model in a two-way repeated measures configuration to assess change over time. Treatment effect will be expressed as estimated effect of intervention, along with a 95 % confidence interval and *p* values ≤0.05 for each time point. The Mann-Whitney *U* test will be used for non-normal distributed continuous variables such as QUALID [49], QUALIDEM [50], NPI-NH [52], CMAI [53], CSDD [54], MMSE [41], MOBID-2 Pain Scale [55], CGIC [59] and P-ADL [57]. Cost-utility analysis will be performed including costs for pharmaceuticals, resource use in NHs and use of external heath care facilities [44]. A full statistical analysis plan, including potential missing data imputation for each outcome measure, will be developed through the course of the study.

### Sample size analyses

QoL is our primary outcome measure, and ongoing comparable intervention studies conduct similar sample size analyses; however, we are not aware of large-scale studies presenting the effect of a multicomponent intervention on QoL outcome measures such as QUALIDEM or DEMQOL. Based upon the magnitude of improvement in our previous RCT on pharmacological interventions [15] for neuropsychiatric symptoms in NH patients, we estimated that a 25 % reduction of the NPI-NH scale (SD 5 standardized effect size [SES] 0.4) for comparison of the intervention and control group at month 4. To measure a difference of this magnitude requires a minimum of 81 patients allocated to each arm of the trial, for a significance level of 5 % (two sided), a power of 80 % and equal allocation. As cluster designs lead to loss of power [47], the sample size should be multiplied by 1 + (*m* − 1)*ρ*, called the design effect, where *m* is the average cluster size and *ρ* = s2b/(s2b + s2w) is the ICC, where s2b is the variance between clusters, and s2w is the variance within clusters. Based on additional assumption of an estimated ICC of 0.157 in the earlier trial with an intervention over 8 weeks [15], an average of 10 eligible patients in each cluster gives a DE = [(1 + (11 − 1) × 0.157)] = 2.57 [15]. Thus, we need a minimum of 208 (2.57 × 81) patients per arm, or 416 patients in total. We expect a drop-out rate between 20 to 25 % [64] from baseline to month 4. Thus, we need a recruitment of an additional 104 participants (520 in total), with 32 clusters (NH units) in each arm. COSMOS will be conducted in at least 64 NH units (clusters), with an average of 8–12 patients on each unit.

### Ethical approval

The trial is approved by the Regional Committee for Medical and Health Research Ethics, West Norway (REK 2013/1765), and registered at clinicaltrials.gov (NCT02238652). Verbal and written informed consent was obtained in direct conversations with all cognitively intact patients with sufficient ability to consent. In patients lacking the ability to consent, verbal and written informed and presumed consent was obtained in direct conversation with the patient (if possible) and his or her legal guardian, usually a family member or advocate, after explaining the aims and protocol of the study.

## Trial status

The trial is an ongoing project; we have completed the pilot, included participants in the trial and now commenced implementation of the COSMOS intervention and data collection at baseline and follow-up, at the time of manuscript submission.

## Discussion

COSMOS intends to improve the QoL in NH patients by enhanced communication and ACP, systematic assessment and treatment of pain, medication review and organization of meaningful activities provided by educated NH staff. Thereby, the intervention aims to improve the mental and physical health of the people, safety and cost-effectiveness and reduce unnecessary medication and hospital admission.

The development of the multicomponent approach is built on evidence-based research results of single-intervention studies. In fact, this intervention was inspired by prompt feedback from NH staff in response to our pain research: “of course, it is important that NH patients are pain free, but our problem is not primarily the pain, but rather the communication—we do not talk to them, early enough” or “nice with less medication, but our problem is lack of activities”. Our research team realized that complex health challenges in NHs are in need of complex and multifaceted and systematic interventions.

In the absence of a comparable study design, the length of a 4-month period is based on current results by a trajectory study demonstrating a death rate of 29 % during the first year after NH admission (submitted). Based on the 2-month pilot study, we recognized that the NH staff needs enough time to get familiar with the COSMOS intervention, teach new colleagues and make necessary changes in the unit. On the other hand, the study period should not be too long, to avoid patient drop-out and ensure staff compliance.

This study design has its limitations. We are aware that the combined COSMOS components into a complex intervention investigated with a cluster randomized research design make the trial more impractical and objectionable compared to a single intervention [65]. It has previously been described that the complexity resulting from interactions among many component parts decreases the predictability of effects [66]. Despite this limitation, we argue that the combination of several components to a multifaceted intervention is necessary to cover a larger area of unmet needs in NH patients and people with dementia. In addition, we suggest that the concept may mimic the clinical reality. To deal with this methodological challenge, we followed recommendations by the implementation science for development and testing of multicomponent healthcare interventions [67]. It has previously been highlighted that the development, implementation and evaluation of any new and systematic healthcare intervention are complex procedures [60]. To avoid study complexity and unpredictability, researchers usually reduce study designs to one of the most essential parts in order to fulfill strict RCT requirements [67]. This reduction may result in a complex intervention being reduced to a series of simple interventions; doing so fails to acknowledge that a complex intervention has the potential to be more than the sum of its parts. Optimistically, results of this study will demonstrate the efficacy of this intervention and satisfaction in patients, staff and relatives. In addition, we expect to contribute to further development of implementation research in the NH setting.

It is also widely recognized [47] that RCTs are less efficient, in a statistical sense; compounded by the effect of personal interactions among cluster members who receive the same intervention. For example, education strategies provided during teaching lessons could lead to sharing of information that creates a cluster effect. Circumstances have an impact on sample size analyses and the necessary volume of the study. Attempts to minimize contamination were made (e.g. geographical distance between NH units and same physicians do not serve different units of control and intervention groups). In addition, we have included a larger group of patients and clusters in accordance with the sample size analysis adjusted for the ICC effect. Until now, there are few comparable studies: The WHELD study [68] includes even more participants; however, the intervention method uses a grid design with different intervention approaches resulting in increased sample size.

Taken together, several structural factors may influence the implementation process and outcome measures [38]. Although much is known about the effectiveness of interventions that benefit aspects of physical and mental health, any intervention is of limited value unless it is practical and can be implemented routinely in clinical practice [37]. Research is imperative to understand and evaluate potential obstacles to refine interventions and competence improving programs through extensive field testing.

## Appendix

**Table 2:** 2-day education program for COSMOS ambassadors, physicians and nursing home managers

	Themes
Day 1	
08:30	Registration, welcome and introduction of participants and nursing homes (NH)
09:00	The multicomponent concept of COSMOS, introduction and plan for teaching, education and follow-up of patients, relatives, NH staff including managers
09:45	Module 1: Assessment and treatment of pain
	Pain physiology; pain behaviour in people with dementia; stepwise protocol of treatment pain
10:30	Break
10:45	Efficacy of treating pain on neuropsychiatric symptoms in people with dementia
11:15	Practical exercises in the use of MOBID-2 Pain Scale; introduction of the manual and demonstration material for the cluster/NH unit
12.15	Lunch
13.00	Module 2: Organization of activities
	What is the evidence base for different types of activities
13:45	How to assess the efficacy of activities?
14:30	Break
14:45	The patient’s individual plan
15:30	Practical exercises in identification of the resources in my NH; introduction of the manual and demonstration material for the cluster/NH unit
16:15	Feedback and evaluation of the day
16:30	Take home message
Day 2	
08:30	Welcome and coffee
08:45	Module 3: Medication review
	Polypharmacy in elderly people and NH patients with and without dementia
09:30	Anticholinergic side effects; START and STOP criteria; www.interaksjoner.no
10:15	Break
10:30	Use of the medication review checklist and relevant patient tools
11:15	Practical exercises of medication review by patient examples; introduction of the manual and demonstration material for the cluster/NH unit
12:15	Lunch
13:00	Module 4: Communication in form of advance care planning (ACP)
	What do we know about ACP and communication in NH settings?
13:45	How to assess the efficacy of ACP?
14:30	Break
14:45	Role play
15:30	Practical exercises in identification of promoters and barriers to conduct ACP in my NH; introduction of the manual and demonstration material for the cluster/NH unit
16.15	What are the next steps? Contact with patients and relatives, telephone hotline, information posters/pocket cards, flyers, contact with media, web-site and more.
16:30	Program evaluation
17:00	Take home message

## Abbreviations

ACP: advance care planning
ADL: activities of daily living
ANCOVA: analysis of covariance
CGIC: Clinical Global Impression of change
CMAI: Cohen-Mansfield Agitation Inventory
COSMOS: COmmunication, Systematic assessment and treatment of pain, Medication review, Organization of activities, Safety
CSDD: Cornell Scale for Depression in Dementia
EQ-5D: European Quality of Life-5 Dimensions
FAST: Functional Assessment Staging Tool
MMSE: Mini-Mental Status Examination
IMMPACT: Initiative on Methods, Measurement and Pain Assessment in Clinical Trials
ICC: intraclass correlation coefficient
MOBID-2: Observation-Behavior-Intensity-Dementia-2 Pain Scale
NH: nursing home
NPI-NH: Neuropsychiatric Inventory-Nursing Home version
NPS: Neuropsychiatric symptom(s)
NSD: Norwegian social Science Data Services AS
RCT: Randomized Controlled Trial
REK: Regional Committee for Medical and Health Research Ethics
RUD-FOCA: Resource Utilization in Dementia-Formal Care
QoL: quality of life
QUALID: quality of life in late-stage dementia
QUALIDEM: quality of life in dementia
SAE: serious adverse event
SD: standard deviation
SPSS: Statistical Package for the Social Science
STOPP: Screening Tool of Older Persons’ potentially inappropriate Prescriptions
START: Screening Tool to Alert doctors to Right Treatment
UoB: University of Bergen
